# CISBETI 2019 - International Congress of Health, Well-Being, Technology and Innovation: Foz de Iguaçu, Brazil. 4-6 April 2019

**DOI:** 10.1186/s12919-019-0166-9

**Published:** 2019-07-24

## A3 Factors related to odor in malignant fungal wounds: Integrative review

### Thalyta C Martins, Patrícia O Salgado, Cristiane C Souza, Laylla M Souza, Silvia A Cardoso, Luciene M Braga

#### Federal University of Viçosa, Minas Gerais, Brazil

**Correspondence:** C. Engel (thalyta.martins@ufv.br)

**Introduction**

Malignant fungal wounds are characterized by the infiltration and proliferation of neoplastic cells in the skin, causing tissue integrity rupture. The prevalence of these lesions is between 5% to 10% [1]. The odor of these wounds is the cause of social isolation for the patient, because it represents a barrier to being close to people, worsening of nutritional state associated with episodes of nausea and vomiting caused by a fetid odor and consequent loss of quality of life. There is a gap with respect to the knowledge of what factors are most relevant in the development of the odor.

**Objective**

To identify in literature the main factors related to the etiology of the odor of malignant fungal wounds.

**Method**

Integrative review of literature. We used the acronym PICO to draw up strategies for searches in the search portal PubMed, CINAHL and LILACS. Inclusion criteria: full articles published in Portuguese, English and Spanish; central theme factors related to the odor of fungal malignant wounds; performed with adult populations; published in the last ten years.

**Results**

The sample of this review was composed by six studies published between 2009 and 2018. Three studies were quasi-experimental (50%), two case reports (33.3%), and a prospective cohort (16.6%). The six articles were published in the English language, having originated from Japan (33.3%), Canada (16.6%), France (16.6%), England (16.6%) and the United States (16.6%). The factors related to the fungal odor in malignant wounds were the infection caused by aerobic and anaerobic organisms, devitalized tissue and the presence of purulent exudate.

**Conclusion**

The knowledge of the factors related with the odor of fungal malignant wounds will allow the development of more effective nursing care, focused on odor control, providing a better quality of life to the patient.

**Keywords**

Wounds and Injuries, Carcinoma, Odorants, Etiology.

**Reference**

1. Tilley C, Lipson J, Ramos M. Palliative wound care for malignant fungating wounds: holistic considerations at end-of-life. Nurs Clin North Am. 2016 Sep; 51(3):513-31. doi: 10.1016/j.cnur.2016.05.006

## A17 Risk areas for infant tuberculosis in a municipality in the interior of São Paulo, Brazil, 2006-2017

### Thais Z Berra, Yan M Alves, Luana S Alves, Ivaneliza S Assis, Marcos A Arcoverde, Antonio Carlos Ramos, Ricardo A Arcencio

#### University of São Paulo at Ribeirão Preto College of Nursing, São Paulo, Brazil

**Correspondence:** Thais Z Berra (thaiszamboni@live.com)

**Introduction**

Tuberculosis (TB) remains a serious global public health problem and accounts for 130,000 deaths per year in children and an estimated 1 million TB cases in the world's child population, TB is one of the top 10 causes of child deaths in the world. The major challenge related to Childhood Tuberculosis (CTB) is its diagnosis. Diagnostic techniques classically used in adults have low sensitivity and specificity in children, and confirmation by bacteriological identification is not always possible.

**Objectives**

The objective of the study was to identify risk areas of TB in children from 2006 to 2017.

**Methods**

An ecological study was carried out in Ribeirão Preto-SP. The study population consisted of all cases of TB in children younger than 15 years of age reported in the TBWeb between 2006 and 2017. The notified cases were geocoded and, for the detection of the areas of spatial risk of children's TB cases, the software was used SaTScan version 9.4 with application of statistical scanning technique. The thematic map was built using ArcGis software version 10.5.

**Results**

96 cases of CTB were reported, 90 cases were geocoded (93.75%). A spatial risk area (p = 0.006) was identified through the scanning statistic, presenting RR = 3.14 (95% CI = 2.05-4.84) involving 167 census tracts in the Central, West and South districts, 33 cases of CTB, population of 17794 children under 15 years.

**Conclusions**

Through the Scan Statistics, it was possible to identify a high risk area for CTB in the Central, West and South districts where it is possible to observe areas with high proportion of residents per household, low Human Development Index and greater concentration of favelas. In this way, the detection of risk areas becomes important for health surveillance and, when applied to spatial data, focuses on the ability to detect changes in the risk of acquiring or dying from a given disease over time and space.

**Keywords**

Childhood Tuberculosis; Spatial Analysis; Scan Statistics; Risk areas.

## A20 Comparison of the Physiological Cost Index between two types of walker in the walking of elderly people with mobility limitations

### Arménio Cruz^1^, Montenegro Lucia^1^, Filipa Couto^1^, Cândida Malça^2^, Luís Roseiro^2^, Pedro Parreira^1^, João Apóstolo^1^

#### ^1^Health Sciences Research Unit: Nursing (UICISA:E) Nursing School of Coimbra, Coimbra, Portugal; ^2^Polytechnic Institute of Coimbra - Engineering Department, Coimbra, Portugal

**Correspondence:** Arménio Cruz (acruz@esenfc.pt)

**Abstract**

**Background**

The growing population ageing and an increase chronic conditions is reflected in the elderly dependency, namely to walk as they are more and more in need of support tools to help them.

**Objective(s)**

Comparing the level of a physiological cost of using a two wheel walker and self-locking kit (AK) with the rigid walker (AF) in elderly with movement limitations.

**Methods**

An almost experimental study, a before-after design with the same group, with a non-probability sample by convenience (n=18), of elderly who walk with rigid walker and are institutionalised in a Home or a Long-Term Care Facility. The data were collected through the measurement and evaluation of the duration, gait speed and physiological cost index during the Expanded Timed Get up and Go Test (ETGUG).

**Results**

A marginally significant difference in the physiological cost index when using the two support products, where it tends to be lower with the use of AK (Z = -1,82; p = 0,068). A relationship between the IMC and the variables of the functional tests was observed.

**Conclusions**

The prescription of a walker should take into account their characteristics and the user, namely IMC. It is suggested that randomized efficacy studies with the introduction of new variables, that contribute to the development of new products to support tools and improve the quality of life.

**Keywords**

Elderly; Walker; Physiological Cost Index Rehabilitation

## A32 Indicators of anxiety in view of the death of university students in the São Paulo Hinterland/Brazil

### Fernando L Macedo^1^, Loiane L Santos^1^, Randolfo S Junior^2^, Patricia M Cury^3^, Júlio C André^2^, Edilson C Caritá^4^, Silvia S Silva^4^

#### ^1^Municipal Institute of Higher Education IMES Catanduva, São Paulo, Brazil; ^2^Faculty of Medicine of São José Rio Preto, São Paulo, Brazil; ^3^Faceres, São José do Rio Preto, Brazil; ^4^Ribeirão Preto University, Ribeirão Preto, Brazil

**Correspondence:** Fernando L Macedo (fernando.planetasurf@gmail.com)

**Introduction**

From ancient times the relation between people and death has been permeated by social, cultural and historical influences. At present there is a significant gap regarding themes associated with death in Brazilian higher education.

**Objective**

To evaluate the attitude of students in different fields of specialization concerning death and dying.

**Methods**

304 university students have been invited from Higher Education Institutions in the municipality of São José do Rio Preto – SP, Brazil, attending medical school 79 (25.99%), civil engineering 78 (25.66%), business administration 81 (26.64%), and psychology 66 (21.71%), who responded to the instrument for sociodemographic description and the revised Collet- Lester scale for fear of death. The sociodemographic data were subjected to descriptive statistics, and the Mann-Whitney and Kruskal-Wallis tests (p< 0.05) were used to compare results across the groups.

**Results and Conclusion**

The sample was comprised of students at an average age of 22.3 years (±5.28). Out of the total participants, 54.6% were female, predominantly single (82.6%), catholic (54.6%) and in the A economic tier (37.5%), according to the Brazilian Economic Classification. Results show, as concerns fear of death, high indicators 95.94% (±22.89) among female participants with a significance value (*p*<0.001) and lower indicators 79.92% (±26.19) among civil engineering students when compared to those in other programs (p<0.001); showing a greater trend toward anxiety before death or other people’s dying, when compared to own death/dying in the sample. The study underscores the relevance of studying death, showing that suffering due to loss is universal. The results are discussed attending to the implications of health programs in delivery of care. The male gender and the choice for programs in exact sciences are protective factors regarding fear of death, thus suggesting that new studies are required to explain the factors influencing the data, which suggest that cultural factors may be at work.

**Keywords**

Behavioral Assessment Scale; Death; Health Psychology; Students.

## A39 Violence against teenagers and coping strategies: An integrative review

### Vanessa S Farias, Maristela I Vasconcelos, Gabriel P Maciel, Yanka A Cavalcante, Milena M Vieira, Paulo R Lopes, Lourival G Júnior, Ana S Cavalcante

#### Estadual University of Vale do Acaraú (UVA)

**Correspondence:** Vanessa S Farias (nessinhasf@hotmail.com)

**Introduction**

Violence is historical and it is always a reflection of the society that reproduces it, and can increase or decrease according to its social construction at the collective and individual levels. Understanding violence as a phenomenon that has affected adolescents around the world in an increasingly frequent and varied way, it is essential to develop studies that characterize violence against adolescents, as well as existing coping strategies.

**Objective**

The purpose of this study was to identify and describe the types of violence that most affect adolescents, the places where they occur, as well as coping strategies

**Method**

It is an integrative review of literature, performed from august to october in 2018, in the databases of the BVS, LILACS, BVS Adolec Brasil and PubMED, with the keywords from the MESH terms: adolescent, violence, public health and school health services, considering the following guiding question: What types of violence are the most affected by adolescents, what makes them vulnerable to these types of violence and what are the coping strategies implemented?

**Results**

The sample was consisted of 144 articles, which were organized and it was characterized according to the year, type of study, types of violence against adolescents, coping strategies and levels of evidence. Bullying, sexual violence and intrafamily violence have emerged as the main types of violence affecting the teenagers. Among the coping strategies are the identification of vulnerability factors and the implementation of intersectoral programs to prevent violence.

**Conclusion**

The study brings contributions to identify the main types of violence suffered by teenagers nowadays, the places where the youth are most vulnerable and offers suggestions for interventions. Being the school environment conducive to situations of violence, the network action articulating the health and education sector were pointed as important components of strategies to combat violence against adolescents.

**Keywords**

Adolescent; Violence; Public health; School health services.

## A40 Social representations of old adults in future social work professionals

### Maria C Faria, Ana I Fernandes

#### Beja Polytechnic Institute

**Correspondence:** Maria C Faria (mcfaria@ipbeja.pt)

**Background**

When considering the professionals involved in the ageing process, we see that they have a determining role in the good conduct of problem-solving, so it is important to know their view about the elderly in these circumstances. In this sense, it is necessary from an early stage to understand the representations and the formative needs of those who embrace a profession. The main objective of this study was to know the perspectives of future social work professionals about older people and, consequently, to identify their social representations about old age and the formative needs to work with this target group. Secondly, we intend to present formative guidelines of ageing.

**Methods**

The Focus Group Technique, a technique for qualitative research, was used. A guide was created that aimed to know the social representations in seven aspects: positive/negative images of the elderly people; contact/acquaintance with elderly people; elderly; ageing; ageing gains and losses; work of the social work professional with the elders of society; and training needs on gerontology. The participants were six students (Women = 4, Men = 2), one of each year of the degree, unmarried, aged between 19 and 26 years (Mean = 21.33 years, SD = 2.11). Sampling was non-probabilistic of the snowball type. Data were analyzed thematically and with descriptive statistics.

**Results**

Findings show that the future professionals reveal understanding about successful (56,24 % of positive evocations) and unsuccessful ageing. They identify the losses and the gains and present positive stereotypes of the elderly of the two genders. However, the participants present a predominantly negative perspective of the ageing (73,48%). They consider that it should be included in the professional training more work in elderly intervention; a discipline of psychology of the elderly; and the implementation of volunteering of students of higher education in the area of ageing.

**Conclusions**

This study aimed to know the perspectives of the future social work professionals on ageing and older people, to identify their social representations about old age and the formative needs to work with this target group. According to the results, training guidelines were drawn up to enable future professionals to practice consciously promoting the quality of life of the elderly in the community.

**Keywords**

higher education students, social worker professional, ageing, adult development elderly society, successful ageing, gerontology, formative guidelines of ageing

## A41 Innovation in Nursing: Development of a double-chamber syringe using human-centered approach

### Liliana B Sousa^1^, Inês Marques^1^, Anabela Salgueiro-Oliveira^1^, Arménio Cruz^1^, Sara Cortez^2^, Filipa Carneiro^3^, Pedro Parreira^1^

#### ^1^Health Sciences Research Unit: Nursing (UICISA:E) Nursing School of Coimbra, Coimbra, Portugal; ^2^Muroplás Plastic Engineering, Muro, Portugal; ^3^PIEP Innovation in Polymer Engineering (University of Minho), Braga, Portugal

**Correspondence:** Liliana B Sousa (baptliliana@esenfc.pt)

The Human-Centered model are widely accepted in order to accomplish the ISO standards. This is an iterative process that involves end users in the Medical Devices (MD) development process, whose main purpose is to obtain successful products that meet user requirements, increasing the usability of a device and user satisfaction.

The insertion of a peripheral intravenous catheter is the most frequent invasive procedure performed in nursing clinical practice, which enables the intravenous administration of fluids/medications on the bloodstream. Flushing the catheters is the most important factor in preventing complications.

The DUO Syringe Project (POCI-01-0247-FEDER-017604) main purpose is the development of a double-chamber syringe that enables the filling and administration of medication and flush solution for use in health services.

We describe the initial steps on the development of a double-chamber syringe that enables the filling and administration of medication and flush solution. Specific methodological procedures were already implemented along the product development cycle: identification of the user, their requirements and context of use; production of design solutions; and pre-clinical evaluation in terms of usability and ergonomics.

Focus groups with users (nurses) were considered in several stages of the product development and some major contributions were made for the improvement of the final design solution: (i) related to syringe plunger (e.g. with different colors to visually differentiate the medicine chamber and the flush solution chamber); (ii) body (e.g. enhancement of the support flaps for the index and medium fingers on the syringe body); and (iii) chambers (e.g. the filling and administration sequences). Also, important recommendations were done, for example regarding the need to ensure that the two chambers were used for their intended purpose (i.e. one chamber for medication and another chamber to the flush solution).

This user-centered approach within the MD design and development provide crucial data for the MD development by industrial and technological partners involved in the double-chamber syringe.

**Keywords**

Syringe, Double chamber, Innovation, Flushing, Human-Centered model, Medical Devices

## A42 Is there a right volume for catheter flushing? Laboratory experiments to predict an optimal volume

### Inês A Marques^1^, Paulo Costa^1^, Ana C Santos^2^, Liliana B Sousa^1^, Arménio Cruz^1^, Vanessa Cortes^3^, Anabela Salgueiro-Oliveira^1^, Pedro Parreira^1^

#### ^1^Health Sciences Research Unit: Nursing (UICISA:E) Nursing School of Coimbra, Coimbra, Portugal; ^2^Biophysics Institute, Coimbra Institute for Clinical and Biomedical Research (iCBR), FMUC; ^3^Nursing department, Federal University of São João del-Rei

**Correspondence:** Inês A Marques (inesafmarques@esenfc.pt)

**Introduction**

Peripheral intravenous catheters (PIVC) are widely used in hospital settings, being estimated that 30-80% of patients will be subject to this invasive device during their hospitalization. Unfortunately, PIVCs are related to a high number of complications, such as phlebitis, infiltration, occlusion, and microbial colonization, the later which could progress to bloodstream infections. Flushing the catheters to remove intraluminal debris and fibrin deposits is described by international guidelines of the Royal College of Nursing as a standard practice that lowers the incidence of such complications. However, although flushing is recognized as an essential procedure to maintain PIVCs’ patency, there is no general consensus on the optimal volume and flushing technique to be used by health professionals.

**Objectives**

To evaluate, through laboratory experiments, the optimal flushing volume needed to maintain PIVC’s patency and eliminate intraluminal deposits.

**Methods**

In order to simulate the normal conditions in clinical settings, catheters were filled with fibronectin (5μg/mL), a blood protein that promotes adherence to the PIVC lumen. Catheters were then left to dry overnight at 37°C. Subsequently, a human albumin solution (50mg/mL) was added to the PIVCs. Catheters were then flushed with 10 mL of saline solution (0.9%) and aliquots of 1 mL were collected. The number of proteins removed from the lumen was measured by spectrophotometry, using the biuret method.

**Results**

The majority of the proteins were removed in the first 1mL collected (more than the double of albumin administered). In the next aliquots collected, approximately the same quantity of protein was removed in each 1mL. Nevertheless, a small amount of protein was still retained inside the lumen of the catheters after the 10 mL flushing.

**Conclusions**

These preliminary results demonstrate that a significant percentage of deposits are removed with a flushing technique with a volume corresponding to twice the PIVC’s intraluminal volume. These findings are in line with the minimum flushing volume stipulated in some of the international recommendations consulted. Nevertheless, given that a lesser amount of proteins was still retained inside the PIVCs, more laboratory tests are necessary to confirm an optimal flushing volume. Further tests should also focus on the type of flushing technique used.

**Keywords**

Peripheral intravenous catheters, adherence, optimal flushing volume, spectrophotometry, laboratory experiments, catheter intraluminal deposits.

**Funding**

Syringe Duo Project (POCI-01-0247- FEDER- 017604)

## A44 Peripheral venous catheter characteristics and materials: A scoping review protocol

### Rafael Bernardes, Anabela Salgueiro-Oliveira, João Graveto, Liliana B Sousa, Paulo Costa, Inês A Marques, Pedro Parreira

#### Health Sciences Research Unit: Nursing (UICISA:E) Nursing School of Coimbra, Coimbra, Portugal

**Correspondence:** Rafael Bernardes (rafael.alvesbernardes@gmail.com)

**Introduction**

Globally, it is estimated that 70% of all hospitalized patients require, at least, one peripheral intravenous catheter (PIVC) to achieve their clinical goals. However, PIVCs are associated with high complication rates, posing a significant risk to effectiveness, quality and safety of the care provided. These adverse events undermine the patient well-being and exponentially increases the costs associated with care provision.

Currently, a significant number of studies addressing such challenge focus on health professionals’ practices during PIVC management; however, recent interest has been raised concerning the impact that current PIVC materials and design may have in the incidence of such complications. Nevertheless, there are no known studies that map all the available evidence on current PIVC materials and design and associated complications.

**Objectives**

To map all the available evidence on PIVC materials and design as well as any described inherent complications.

**Method**

A scoping review protocol was established, following Joanna Briggs Institute recommendations. A three-step search strategy will be used to identify both published and unpublished studies: i) An initial limited search in MEDLINE (via PubMed) and CINAHL complete (via EBSCO), followed by an analysis of text words in titles and abstracts and index terms used to describe the articles; ii) A second search using all identified keywords/index terms across the included databases; iii) The reference list of all identified articles and reports will be analyzed to identify additional studies. Studies written in English, Spanish, French, and Portuguese will be considered for inclusion in this review, regardless of the year of publication. Two independent reviewers will assess all articles for relevance, as well as perform data extraction and synthesis.

The initial search strategy will use the conjugation of the following terms: catheter*; cannula*; “vascular access device”; "peripheral intravenous catheterization"; “"peripheral venous catheterization"; characteristic*; material*; “adverse effect”; complication*; fail*.

**Conclusion**

Presently, a small number of studies are focused on PIVC materials and design as a potential cause of catheter failure. This review is expected to map all known contributions in the literature, supporting the potential development of innovative catheters that can mitigate these occurrences and contribute to PIVC-related effectiveness and safety.

**Keywords**

catheters; medical devices; vascular access devices; scoping review; adverse effects; complication; cannula.

## A45 The importance of the family unit in the social reinsertion of the assistants of the Basta group: Municipal penitentiary patronage of Foz do Iguaçu-PR

### Esther Silva, Ewandro Luiz, Mónica Mombeli

#### University Center Dinâmico das Cataratas, Foz Iguaçu, Brazil

**Correspondence:** Esther Silva (estherjcsilva@gmail.com)

The present study aims to investigate the importance of the family nucleus in the social reintegration of the BASTA assisted group that was sent through the Criminal Execution Court of Foz do Iguaçu-PR to the Municipal Penitentiary Patronage. The referral is made by the magistrates according to the Municipal Law (LEI N° 4085, OF MAY 6, 2013). Semi-structured interviews were carried out with professionals in psychology, pedagogy and social workers to collect data. In this way it is understood that in the constitution of a subject, the link with the family is of great importance. It is through the family that are constituted values and behaviors that respond as acts of civil life traditional, geographic and socioeconomic factors interfere in the judgment and the decisions that individual assumes before the society that is inserted, as is provided in law there are guarantees, duties and punishments those who do or break it. The problem that involves the formation of the subject can’t be anticipated, it is only with the connection established since the earliest childhood stages that the first experiences with socializing and with the participation of the family network are created, thus drawing great contribution in the personality of the citizen and his vision of the world.

**Keywords**

Subject, Family, Negligence, Development.

## A46 Identification of low back pain risk in industry employees by functional thermography performance

### Pitiguara Coelho^1^, Pâmela R Vidal^1^, Bruna C Lyrio^1^, Alvaro R Junior^1^, José L Ramos^1^, Hebert W Cabral^2^, Marcos L Brioschi^3^

#### ^1^Instituto GVIX, Vila Velha, Espírito Santo, Brazil; ^2^SME Consultoria, Vitória, Espírito Santo, Brazil; ^3^Faculdade de Medicina da Universidade de São Paulo, São Paulo, São Paulo, Brazil.

**Correspondence:** Pitiguara Coelho (pitiguara@gmail.com)

**Introduction**

Low back pain, a condition that affects both genders, with a greater incidence on young adults in an economically active phase is common among adult workers. Aiming at the emerging needed for identification of these cases, infrared thermography is a highlight, which is an effective and low-cost tool for the evaluation of pathologies that affect the musculoskeletal system.

**Objectives**

To evaluate lumbar and thoracic thermography associated with functional incapacity of low back pain in workers.

**Method**

A cross-sectional study carried with 85 employees from the administrative and operational sectors of an automotive multinational. For the data collection of the infrared thermography, the Functional Thermokinetic Perfomance (PFT) was used a methodology that consists of the thermographic analysis using a thermographic diagnostic equipment, having a personalized analysis system, which classifies the individual's risk for the development of low back pain. In addition to the diagnosis, the PFT has a therapeutic intervention plan aimed at improving the patient's condition and reducing the risk of low back pain. In the study, phase I was used, which corresponds to the thermographic diagnosis. The Roland-Morris low back incapacity questionnaire was also applied. For statistical analysis, we used SPSS software version 23 and the Pearson's Chi-squared and Kruskal-Wallis tests.

**Results**

The sample was composed mainly of men (87.1%), with ages ranging from 28 to 49 years (77.7%), mainly from the operational sector (68.2%), with service time of five years or more (51.8%). The mean score of Roland Morris was considered low risk (2.82); however, the PFT risk score was 38.8% for high risk and 36.5% for very high risk. Comparing the variables, was observed a statistical association with the female gender and the ratings of high and very high risk. Time of service, age range, and work sector did not present significant statistical correlation.

**Conclusions**

The study results suggest that the PFT can more effectively predict the risk for low back pain. Although women presented higher risk, it is noted that the risk for functional incapacity of low back pain is independent of age, sector and length of service, showing that this risk is equivalent for both.

**Keywords**

Back Pain, Thermography, Worker Health.

## A48 Psychosocial risks in work at height: Perception of professionals

### Mónica Mombelli, Bruna Pierezan, Pamela Potratz, Mayara Kastring

#### University Center Dinâmica das Cataratas, Foz Iguaçu, Brazil

**Correspondence:** Mónica Mombelli (psicmonicamombelli@gmail.com)

Psychosocial risks represent a set of perceptions and experiences that include the modifications and interactions of the person in the occupational environment, in addition to their personal characteristics, their culture, their necessities, their lifestyles, their conceptions of the world that influence social, economic, health and safety at work, causing physical, social and mental damages to the employee. The present study was performed with 30 male workers, whose main objective was to identify the psychosocial risks related to work at height. It is a qualitative study and, to collect the data, a questionnaire was used by the authors. The information collected was analyzed through IRAMUTEQ software which permits the analysis of textual data. The results show that non-occupational psychosocial risks are related to situations experienced in the family and occupational psychosocial risks refer to interpersonal relationships in the work context. In addition, the perception of workers on the importance of safety at work is noted. His speeches reveal that safety is focused on the use of safety equipment, standards and training, not addressing aspects of mental

**Keywords**

Mental Health. Psychosocial risks. Work at height.

## A49 What is the real importance of flushing for intravenous therapy? Preliminary data

### Pedro Parreira^1^, Grazielle Maia^2^, Duane Pereira^2^, Inês Marques^1^, Paulo Costa^1^, Anabela Salgueiro-Oliveira^1^, Daniela Mendonça^2^,Vanessa Cortes^2^

#### ^1^Health Sciences Research Unit: Nursing (UICISA:E) Nursing School of Coimbra, Coimbra, Portugal; ^2^Nursing department, Federal University of São João del-Rei

**Correspondence:** Pedro Parreira (parreira@esenfc.pt)

**Introduction**

Intravenous (IV) therapy is an effective and fast-acting way to administer fluid or medication; however, under-dosing can result in general therapy failure. IV drugs can be administered via bolus through peripheral venous catheters (PVC). Current standard practice advocates for PVC flushing after each single drug administration to prevent catheter obstruction and drug interaction. Nonetheless, very little attention has been paid to identifying the optimal flushing volume needed to clear the PVC lumen.

**Aim**

To determine if the recommend flushing volume is adequate to clear the lumen of a PVC catheter and PVC epicranial needle.

**Material and Methods**

As per normal clinical practice, an epicranial needle and PVC catheter (21G and 20G, respectively) were selected and flushed with a 10ml 0.9% normal saline, and 10 aliquots were collected. A solution of 5g/dl albumin was injected through both PVCs, and another 10ml 0.9% normal saline flush was performed. Subsequently, protein level was determined using two different spectrophotometry methods, Bradford (1976) and Hartree (1972) in all 10 aliquots.

In order to evaluate the efficiency of flushing on intraluminal blood adherence, venipuncture was performed and the blood drawn was washed with 0,9% normal saline. The flushed blood was hemolyzed and hemoglobin was quantified using the Drabkin and Austin (1935) method in all 10 aliquots. To confirm the amount of albumin and hemoglobin an SDS-Page gel 10% was used.

**Results**

Overall, the PVC catheter retained more albumin and hemoglobin. After the 5th aliquot of 0.9% normal saline, the PVC lumen retained 50% of the injected hemoglobin and 25% of the injected albumin. Moreover, a residual amount of protein (approximately 10%) was found in the PVC epicranial needle until the 10th aliquot. Electrophoresis suggested the presence of albumin and hemoglobin in the lumen of the PVC catheter and the presence of only hemoglobin on the PVC epicranial needle.

**Conclusion**

Our experiment suggests that the PVC catheter retains more proteins than the PVC epicranial needle after a standard normal saline flush. This study opens an important research field with potential impact on PVC-related care quality and safety.

**Keywords**

Peripheral venous catheters, administration of intravenous medication, Bradford and Hartree spectrophotometry methods, efficiency of flushing, Drabkin and Austin method.

## A50 Mining of climatological data and urban variables associated to the dengue incidence: Study of case in Iguassu Falls, Paraná

### Gilberto Batista

#### University Center Dinâmica das Cataratas, Foz Iguaçu, Brazil

**Correspondence:** Gilberto Batista (gilbertoensino@gmail.com)

Dengue fever is currently one of the most critical diseases that affect the human beings in the urban area. It is a serious public health problem due to the accelerated urbanization, lack of water supply and of urban cleaning and intensive use of disposable material. These conditions favor the rapid proliferation of the main vector of the disease, the mosquito of the species Aedes aegypti. It has been observed that in Foz do Iguaçu, state of Paraná, Brazil, the number of people affected by the dengue fever has increased in recent years. For this reason, cooperation between governmental authorities and civil society has become essential to prevent the spread of the mosquito and thus to reduce the incidence of this disease. This study aimed the spatial-temporal distribution analysis of cases of dengue fever that occurred during the period of 2010 to 2015 in Foz do Iguaçu by geoprocessing tools and data miner. Data concerning the confirmed number of dengue cases were collected from Municipal Health Secretary, Epidemiological Surveillance Division e Surface Weather Stations of the INMET (National Institute of Meteorology) and ICEA (Institute of Airspace Control) and processed in the geoprocessing with Geographic Information System. The monthly and yearly distributions of cases in the urban area were correlated with pluviometric and elaborate and the prediction model. The presented results showed that the climate conditions of the city, rain, populated areas, flood and geographical conditions favored the development and transmission of dengue fever. The study showed the pattern of spatial distribution of dengue fever that was repeated in the years and in the most affected sectors of the city: west, north and central. The spatial patterns observed can be helpful to predict which sectors may have higher incidence of dengue in the following months. Thus, actions may be adopted for monitoring and control, with the strategy of stratification of areas. One can thus identify the characteristics of the sectors that have a higher risk of contamination and use the most appropriate steps. Techniques of spatial analysis and data miner have the potential for disease surveillance and control of dengue fever.

## A51 Study of the environmental sustainability applied to the urban profile of Iguassu Falls

### Gilberto Batista

#### University Center Dinâmica das Cataratas, Foz Iguaçu, Brazil

**Correspondence:** Gilberto Batista (gilbertoensino@gmail.com)

The sustainability circle is a tool of analysis of the dimensions of environmental sustainability. In the proposed article a study of environmental sustainability of the city of Iguaçu Falls was accomplished in the context of the aspect ecology in relation to the themes emissions and residues of the urban area. The urban profile was outstanding with their respective characteristics and peculiarities, once they are outstanding the dimensions of the environmental sustainability for an effective urban administration. The persistence of the production model and consumption in energy not just degrade the nature, but the conditions of life of the population. A maintainable society supposes that all of the citizens have the necessary minimum for a worthy life and that nobody absorbs goods, natural and energy resources that are harmful to the citizens. The analyzed Information are based on the methodology of the sustainability circle and discussed in order to determine the characteristics of the municipal district with their respective externalidades. The municipal district presents in his/her behavior multidisciplinar the concern in softening the problem caused by the too much generation of residues, however the municipal public administration needs to act in the infrastructure of the sanitary embankments and in the implementation of public politics.

## A52 Microbiological quality analysis of raw salmon used for sashimi preparation in Japanese cuisine restaurants in the city of Foz do Iguaçu – PR (Brazil)

### Manal M Rahal, Juliana V Tasca, Matheus S Matias, Áquila P Gonálves, Júlia R Ottoni

#### University Center Dinâmica das Cataratas, Foz Iguaçu, Brazil

**Correspondence:** Manal M Rahal (manalrahal@hotmail.com)

The Japanese gastronomy is a cuisine which has been gaining consumers all over the world. Among the different types of fish, salmon is one of the most utilized, representing a food of high protein content and a source of omega-3, an essential fatty acid associated to health promotion and disease prevention. The fish intended for raw consumption is considered a risk to human health if contaminated with microorganisms. The Brazilian government establishes the procedures to be followed for prepared food (Resolution RDC n° 216 of 2004) and also defines the microbiological limits for microorganisms in fish, consumed without previous cooking, which includes sashimis (Resolution RDC n° 12 of 2001). In this context, the present work aimed to evaluate the presence of indicator and pathogenic microorganisms in salmon used for the preparation of sashimis in restaurants of Japanese cuisine in the city of Foz do Iguaçu – PR by the technique of multiple tubes. Three samples of 30 g of salmon sashimis, from three different restaurants, ie restaurant “A”, “B” and “C”, were acquired, prepared and tested, in triplicates of 10 tubes each for detection of total coliforms, fecal coliforms, Escherichia coli, positive-coagulase Staphylococcus and Salmonella sp. with the use of specific culture media. The assays for the detection of Salmonella sp. were positive for 100% of the samples from the three evaluated restaurants, while the Staphylococcus assays resulted in 2,049 atypical colonies, being 290, 986 and 773 from restaurant A, B and C, respectively. The assays to detect the presence of coliforms were positive for 46% of the samples, both for total and thermotolerant samples, subsequently confirmed in 15, 10 and 12 replicates from restaurant A, B and C respectively. The results showed a high contamination by microorganisms, including Salmonella sp. All restaurants indirectly had deficiencies in the application of Good Practices inside the salmon chain, from acquisition, storage and handling, until reach the final consumer. As raw salmon products are very perishable products, the establishment offering this fish must strictly follow the resolutions imposed by law in order to ensure a safe food for the population.

## A53 Analysis of the use of proton pump inhibitor drugs in patients with gastroenterological diseases in Foz do Iguaçu, PR – Brazil

### Abir A Hijazi, Carolini Slovinski, Fabiola Z Schuvierski, Jackson L Domareski, Ellen C Baier, Carlos H Schneider

#### University Center Dinâmica das Cataratas, Foz Iguaçu, Brazil

**Correspondence:** Abir A Hijazi (abir_hijazi@outlook.com)

**Introduction**

Proton pump inhibitors (PPIs) are a class of drugs used to treat gastroenterological diseases, the main drugs of this class are Omeprazole and Pantoprazole. PPIs promote the healing of gastric and duodenal ulcers as well as improve gastroesophageal reflux. They are usually used continuously, and their prolonged use is considered safe. However, studies warn of risks related to this, and recommend their use through precise indications and periods established by prescribers.

**Aim**

Analyze the effects of prolonged use of omeprazole and pantoprazole in patients in medical clinics in Foz do Iguaçu, PR.

**Methods**

We analyzed 120 patients in the treatment of gastroenterological diseases, who use these drugs from three private medical clinics in Foz do Iguaçu-PR, through the application of a semi-structured questionnaire. This study was released by the Human Research Ethics Committee (2,884,028).

**Results**

Among the patients analyzed it was possible to verify that omeprazole was the medicine with the highest frequency of use in the 03 clinics. The diseases with the highest prevalence were gastritis and gastroesophageal reflux. Regarding the patients studied, 94% had no side effect during the use of these drugs. Among the most commonly reported side effects are nausea, stomach pain and headache. Regarding frequency of use, 39.81% of patients use daily for protection of the stomach, over a long period, between 3 and 15 years.

**Conclusions**

We found a low rate of side effects in patients who make frequent use of PPIs, perhaps that is associated with their rational use, that is routinely accompanied by a doctor. However, it is worth emphasizing that patients should always be informed about possible adverse effects related to the continuous use of these drugs. In this context, qualified pharmaceutical attention is available in pharmacies to meet the needs and clarify the doubts of the population regarding the use of the drug.

## A54 Tamoxifen as a causing agent of hepatic damage in patients with breast cancer in a reference hospital of the frontier triplice in Foz do iguazu, pr brazil

### Jennifer T Oliveira, Tamires A Maria, Fabiola Z Schuviecerski, Jackson L Domareski, Carlos H Schneider

#### University Center Dinâmica das Cataratas, Foz Iguazu, Brazil

**Correspondence:** Jennifer T Oliveira (jenni.tamires@gmail.com)

**Background**

The breast cancer is a topic of great impact on global public health, due to its progressive development in women of different age groups and different causes of death. Various clinical interventions can be performed in the treatment, among them surgical ones accompanied by chemotherapy, radiotherapy, biological treatment and hormone therapy. Medicines used in treatment may reduce the amount of circulating estrogen, such as aromatase inhibitors, or may prevent hormone receptors in tumor tissue, such as Tamoxifen. Tamoxifen inhibits the secretion of VLDL (very-low-density lipoprotein) cholesterol, thus increasing hepatic steatosis.

**Aim**

The aim of this study was to identify possible hepatic damage caused by tamoxifen during the treatment of breast cancer. Methods: Medical records and examinations of 34 women who used this medication between January 2012 and December 2017 were analyzed in a Reference Hospital for the Foz do Iguaçu / PR region. This study was released by the Human Research Ethics Committee (2,884,026). The liver parameters analyzed in this study were the results of ALT (Aspartate aminotransferase), AST (Aspartate transaminase), Bilirubin, alkaline phosphatase and imaging tests.

**Results**

Our data demonstrated that 47.06% of the patients presented hepatic steatosis after tamoxifen and 23.53% presented changes in ALT, AST, bilirubin and alkaline phosphatase. Previous data already demonstrate a high rate of hepatic steatosis in users of this drug. When we categorize our data by age group of patients, we found that the highest rate of liver changes is in women between 41-60 years. Possibly these damages are associated with combinations of factors, including hormone rates, alcohol use or other medications.

**Conclusions**

Although the use of Tamoxifen is indicated in patients affected by breast cancer, it is extremely important to follow these patients with regular liver exams to verify the functions as well as to perform imaging tests in order to verify signs of steatosis.

## A55 Evaluation of the antimicrobial activity of pepper-rosemary essential oil (lippia origanoides kunth), against salmonella sp

### Camila C Castro, Lunara V Peres, Juliana Bilha, Leonardo E Ferreira, Júlia Ottoni, Maria A Toscan

#### ^1^University Center Dinâmica das Cataratas, Foz Iguaçu, Brazil

**Correspondence:** Camila C Castro (camila_cristina98@outlook.com)

Essential oils extracted from aromatic plants have a variety of compounds with antimicrobial activity and represent an interesting alternative for the control of bacteria such as Salmonella sp. The aim of the present work was to evaluate the antimicrobial activity of the essential oil of pepper-rosemary (Lippia origanoides Kunth.), focusing on its ability to control the development of Salmonella sp. Feces were collected from a capuchin monkey (Sapajus apella) contaminated with Salmonella sp. and cultured in Brain Heart Infusion broth for identification and isolation of bacteria. Isolates were further inoculated on agar plates containing Salmonella-Shiguella medium, to identify and isolate strains of Salmonella sp. Rosemary essential oil was obtained commercially and applied at concentrations of 0.1, 0.5, 1, 2, 5% having hydrated cereal ethyl alcohol as solvent. Antimicrobial susceptibility was assessed through the disc-diffusion method. Paper discs were embedded in Rosemary essential oil in each concentration and in the Gentatec antibiotic (gentamycin 40 mg/mL). Four discs of each oil concentration were placed in nutrient agar plates previoulsly inoculated with Salmonella sp., in quadruplicate, and then incubated at 36 °C for 48 hours. The diameters of formed halos were measured using a ruler and results were compares using variance analysis, followed by the Tukey test, with 5% significance. The results indicated that the essential oil at 2 and 5% produced halos with diameter values similar to those of the Gentatec antibiotic (p> 0.05), whereas in the concentrations of 0.1, 0.5 and 1% presented smaller halos compared to that of the Gentatec antibiotic (p <0.05). Results showed that pepper-rosemary essential oil inhibited Salmonella sp. in 2 and 5% concentrations, proving to be a promising agent for the control of this bacteria, thus presenting an alternative in substitution for the use of synthetically produced antibiotics, since the use of essential oils presents lower risks of microbial resistance.

## A62 Impact of the use of three dimensions of a walker in the functional performance profile in institutionalized elderly

### Rosana Silva^1^, Pedro Parreira^1^, Manuel Lopes^2^, César Fonseca^2^, Javier Berrocal^3^, Jaime Galán^3^, Arménio Cruz^1^

#### ^1^Health Sciences Research Unit: Nursing (UICISA:E) Nursing School of Coimbra, Coimbra, Portugal; ^2^University of Évora – Nursing School of Évora, Évora, Portugal; ^3^University of Extremadura, Extremadura, Spain

**Correspondence:** Rosana Silva (rosanamjsilva82@gmail.com)

**Introduction**

Aging is associated with acute and chronic problems that can affect the mobility and independence of the elderly in self-care. Currently, there are several types of walkers on the market, which aim to compensate mobility limitations and fall episodes, however, different performance profiles may be associated with them. For the purpose of the study, a performance profile was defined for institutionalized elderly people, such as time spent walking speed, heart rate variance, and energy expenditure incurred in the institutionalization of the Expanded Timed Up and Go Test.

**Objective**

To compare the performance profile of institutionalized elderly people taking into account the time spent, walking speed, heart rate variance and the energy cost incurred in carrying out the Expanded Timed Up and Go Test in the use of a walker in its three dimensions: fixed, two wheels and four wheels.

**Method**

A quasi-experimental study, pre and post-test, single-group, in institutionalized elderly.

**Results**

When considering the variables of the performance profile when comparing the 3 dimensions of the walker, significant differences were observed in the time covered (X^2^ = 15.80, p <0.001) and walking speed (X^2^ = 15.80, p <0.001). Statistically significant differences were not found in the remaining variables.

**Conclusions**

To the extent that there is no single ideal walker, but personalized to the person's characteristics and needs, it is important to carry out new studies that allow the development and innovation of these devices, as well as the incorporation of new technologies into the competencies of nurses, as is the case of nurses who specialize in rehabilitation nursing.

## A63 The Influence of Psychological Capital and Organizational Culture on the Performance of the Health Organization

### Ana Cristina Oliveira^1^, Lisete Mónico^2^, Anabela Salgueiro-Oliveira^1^, César Fonseca^3^, Damasceno Dias^4^, Pedro Parreira^1^

#### ^1^Health Sciences Research Unit: Nursing (UICISA:E) Nursing School of Coimbra, Coimbra, Portugal; ^2^University of Coimbra, Coimbra, Portugal; ^3^University of Évora – Nursing School of Évora, Évora, Portugal; ^4^ Higher Institute of Social and Political Sciences, Portugal

**Correspondence:** Ana Cristina Oliveira (anacerejo@sapo.pt)

In Portugal, the Primary Healthcare reforms in progress since 2005 aimed to implement a new organizational culture focused on the empowerment and psychological capital of its professionals signaling a bottom-up process. Recent investigations point to the importance of positive psychological capital and organizational culture in organizational performance generating positive operational results with job satisfaction.

Starting from the research question: "To what extent do the psychological capital of professionals based on organizational culture impact their organizational performance?" was performed an empirical, cross-sectional, retrospective, non-experimental, descriptive-correlational study with a sample of 193 health professionals (doctors and nurses) from of 32 health units (Community Care Units, Personalized Health Care Units, Family Health Units) that sought to assess the contribution of psychological capital and organizational culture to organizational performance.

The Psychological Capital scale of Luthans, Youssef and Avolio was used; Organizational Culture scale, by Cameron and Quinn based on the Competing Values Framework; Organizational Performance Questionnaire based on indicators of the internal contracting of ACeS; Job Satisfaction of Brayfield and Rothe; and Intent on Leaving the Organization of Meyer, Allen and Smith. Confirmatory factorial analysis and reliability evaluation through Cronbach's alpha. The hypotheses of correlation analysis, regression models and moderation studies were tested.

The results suggest the existence of a positive relationship between psychological capital, organizational culture, organizational performance and job satisfaction. The regression models show that the US typology explains 5% of the USF/UCSP organizational performance. Organizational culture increases 11% (ΔR2) of organizational performance, specifically due to the Flexibility factor. In total, the model explains 21% of the organizational performance of the USF/UCSP. Psychological capital does not have a predictive effect on organizational performance. However, the mediation studies show a very significant indirect effect: β = .61 * β = .72 = β = .44. in which the organizational culture has a mediating effect. Psychological capital accounts for 38% of the variance in organizational culture that explains organizational performance (β = .72). Organizational culture mediates psychological capital over organizational performance with a global effect of 42%.

The implications of the results obtained are important in the management and training of health professionals given the importance of developing an organizational culture that favours organizational performance. Future research should be directed to the people, employees and users of health services, to evaluate beyond the impact of culture on the performance of the organization, the mediating effect it may have on the effectiveness of the service provided to the citizen.

**Keywords**

positive psychological capital, organizational culture, organizational performance, job satisfaction, organizational commitment

## A64 Umbilical Cord Blood donation process: Motivation and feelings of midwives

### Filipe Lima^1,2^, Tânia Cunha^2^, Beatriz Araújo^3^, Lisete Mónico^4^, Anabela Salgueiro-Oliveira^5^, Aline Marques^6^, Pedro Parreira^5^

#### ^1^PhD Student: Universidade Católica Portuguesa, Porto, Portugal; ^2^Instituto Português do Sangue e Transplantação, Porto, Portugal; ^3^Centre for Interdisciplinary Research in Health (CIIS): Universidade Católica Portuguesa, Porto, Portugal; ^4^University of Coimbra, Coimbra, Portugal; ^5^Health Sciences Research Unit: Nursing (UICISA:E) Nursing School of Coimbra, Coimbra, Portugal; ^6^High Education Institute of Censa, Rio de Janeiro, Brazil

**Correspondence:** Filipe Lima (filipedglima@hotmail.com)

**Introduction**

The Portuguese public cord blood bank (BPCCU) has the mission of making available, to all citizens in need, safe and effective hematopoietic progenitor cells for transplantation, from altruistic donations.

The BPCCU established a protocol with maternity hospitals, which, although physically separated, are fixed collection sites, integrate their functional structure and carry out, recruitment and selection of donors, collection, identification and store of umbilical cord blood (UCB) units.

For each maternity there is a coordinator nurse of the BPCCU team, who is responsible for the implementation of the UCB collection process, professional training, process monitoring, results reporting and motivate the midwives/maternity team.

The UCB donation is a time-consuming process. It involves raising the donor's awareness, completing the maternal questionnaire, obtaining informed consent and performing the collecting procedure. It requires the involvement of the maternity professionals who are focused in delivery, which is reflected in the low collection rates (15%), in relation to the number of deliveries performed.

**Objective**

This study aims to know the motivation of midwives for the UCB collection and to identify the feelings associated with the donation.

**Methods**

The data were obtained through a semi-structured interview, carried out to 10 Midwives. The interviews were previously scheduled, voluntary and anonymous participation requested. Data were recorded, transcribed and analysed using a content analysis technique.

**Results**

The analysis of the data allowed the division of the theme of feelings into two categories: feelings associated with the collection process - negative feeling of overload and positive- gratification; and the feelings associated with utility - solidarity, help.

Professionals feel motivated, but recognize that overwork, high discards and the widespread wear felt in the profession can interfere in this process.

They were identified as strategies to increase motivation: greater presence of the team in the maternity; presentation of results; incorporation BPCCU mission and the possibility of gratifications in service improvements.

**Conclusion**

The results allow infer that professionals recognize their participation in the benevolent and altruistic act of UCB donation, and that in general is associated with positive feelings. Strategic measures to increase motivation have been identified and should be considered to increase the collection rate.

**Keywords**

Umbilical cord blood donation, fellings, motivation

## A65 Two venipunctures in umbilical cord blood collection: Perception and real use by midwives

### Filipe Lima^1^, Tânia Cunha^1^, Beatriz Araújo^2^, Lisete Mónico^3^, Anabela Salgueiro-Oliveira^4^, César Fonseca^5^, Pedro Parreira^4^

#### ^1^Portuguese Institute of Oncology Coimbra, Portugal; ^2^Portuguese Catholic University, Lisbon, Portugal; ^3^University of Coimbra, Coimbra, Portugal; ^4^Health Sciences Research Unit: Nursing (UICISA:E) Nursing School of Coimbra, Coimbra, Portugal; ^5^University of Évora – Nursing School of Évora, Évora, Portugal

**Correspondence:** Filipe Lima (filipedglima@hotmail.com)

**Introduction**

The collection of umbilical cord blood (UCB) is a unique moment, it requires trained professionals with skills to execute the technique, to obtain the maximum blood and an adequate cellular recovery.

In this way, the Portuguese public bank developed a standard operating procedure and disseminated for the execution of the UCB collection technique, to maximize the volume and to minimize the 70% discarded rate.

The established technique is performed after birth with the placenta in utero and consists in obtaining by gravity the blood that remains in the placenta and the umbilical cord, through the execution of two distinct punctures in the umbilical vein (one most distal and other in the proximal region of the placenta).

**Objective**

This study aims to identify the perception that midwives have about the utility of two venipunctures in performing the UCB collection technique and to identify its real use for maximizing blood collected

**Method**

The data were obtained through a semi structured interview, carried out to 10 Midwives. The interviews were previously scheduled, voluntary and anonymous participation requested. Data were recorded, transcribed and analysed using a content analysis technique.

**Results**

Ten midwives participated in the study, 9 females and 1 male. The experience of the participants in the UCB collection ranged from 2 to 20 years.

Concerning the perception of utility, most of the participants do not consider, the execution of 2 venipunctures, useful for obtaining more volume. During interviews it is said that: "There is no need, usually with a puncture is achieved", "a puncture is enough".

Regarding the real use of the procedure only 1 participant reported doing, 4 admit being rare, 3 sometimes and 2 never do.

In the opinion of the interviewees, the second venipuncture is associated with the contamination of unit and the formation of clots.

**Conclusion**

The midwives do not recognize the utility and do not use the second puncture to maximize the volume collected.

The results of this investigation recommend a reflection about the accuracy and assessment of the procedure.

In practice the collection manager should make a quality improvement of these procedures to minimize the number of discards.

**Keywords**

Collection of umbilical corb blood, midwives, venipuncture

## A66 The influence of emotional intelligence while buffer effect on stress

### Catarina Cruz^1^, Lisete Mónico^2^, Anabela Salgueiro-Oliveira^1^, César Fonseca^3^, Damasceno Dias^4^, Carla Carvalho^2^, Pedro Parreira^1^

#### ^1^Health Sciences Research Unit: Nursing (UICISA:E) Nursing School of Coimbra, Coimbra, Portugal; ^2^University of Coimbra, Coimbra, Portugal; ^3^University of Évora – Nursing School of Évora, Évora, Portugal; ^4^ Higher Institute of Social and Political Sciences, Portugal

**Correspondence:** Catarina Cruz (catarinacruz_821@hotmail.com)

**Introduction**

The rise of new technologies and globalization leads tend to a people management valuing the skills of emotional intelligence (EI) expressed. This assumes a positive impact on people management, especially in health care.

**Objective**

The present study aimed to clarify the relationship of IE with the perceived stress (PS) in the management of people, in particular in health professionals.

**Methods**

Integrative review of literature. The question of departure [in relation to healthcare workers (P), how EI acts as buffer effect (I) in PS (C) in your workplace (O)], contributed to the definition of the inclusion criteria. The selection of the articles followed the guidelines according to the PRISMA (Preferred Reporting Items for Systematic Reviews and Meta-Analyses) and indexed in the databases CINAHL, MEDLINE, Nursing & Allied Health, B-On, with a time horizon from 2011 to 2017. The final sample consisted of 14 eligible articles from different countries.

**Results**

The eligible studies reveal that IE presents a moderating effect on the relationship stress/ burnout, with greater evidence in professions that require greater emotional involvement, as health workers. The analysis showed that managers, to make the team your invincible, should adopt strategies that enhance the reduction of PS employee and, at the same time, encourage participation in training programmes for development of EI.

**Conclusion**

We conclude that emotional intelligence can have an impact on the health of organizations, to the extent that employees with higher values of emotional intelligence are more effective to manage your emotions and also the emotions of others, What makes them proactive people and which will enhance productivity-enhancing, as are leaders among their peers.

**Keywords**

Emotional intelligence, perceived stress, people management

## A67 Early maladaptive schemes: The predictive role of parental education and attachment styles

### Lisete Mónico^1^, Paula Silva^2^, Fábio do Prado^3^, Maria São João Brêda^1^, Paulo Pinto^4^, Damasceno Dias^5^, Pedro Parreira^6^

#### ^1^University of Coimbra, Coimbra, Portugal; ^2^ University of Extremadura, Extremadura, Spain; ^3^University Center Dinâmica das Cataratas, Foz Iguaçu, Brazil; ^4^Federal University of Juiz de Fora, Minas Gerais, Brazil; ^5^ Higher Institute of Social and Political Sciences, Portugal; ^6^Health Sciences Research Unit: Nursing (UICISA:E) Nursing School of Coimbra, Coimbra, Portugal

**Correspondence:** Lisete Mónico and Paula Silva

This study investigated the predictive abilities of parenting styles (PS) on early maladaptive schemas (EMS), after mediation of attachment styles (AS) and controlling depressive anxious and stressful symptomatology.

The sample was composed by 450 Portuguese subjects, aged between 18 and 55 years old and participants were evaluated with scales EMBU - one's memories of upbringing, ECR-RS – to evaluate attachment, YSQ-S3 – to evaluate early maladaptive schemas, DASS-21 – for depression, anxiety and stress, and sociodemographic questionnaire.

Results indicated significant correlations between PS, AS, EMS and symptoms of depression, stress and anxiety.

We found significant negative correlations between more positive PS and insecure attachment, and negative PS and all EMS. Insecure attachment correlated with more EMS. Higher symptoms’ scores of depression, anxiety and stress were positively related with more EMS and negative PS. Relations among some PS variables (overprotection e rejection) and EMS were mediated by AS (anxiety and avoidance) after controlling depression, anxiety and stress. Mother’s overprotection often appeared positively linked to EMS, and that relation was mediated, commonly, by depression, predicting nine EMS, mostly belonging to Disconnection Rejection Domain.

The negative PS and insecure attachment predict more EMS and correlate with more symptoms of depression, anxiety and stress. Attachment, depression, anxiety and stress are mediators of the PS and EMS relationship.

**Keywords**

Parenting styles, attachment, early maladaptive schemas

## A68 The Role of Emotional Intelligence in the Organizational Stress and Job Satisfaction

### Lisete Mónico^1^, Hugo Lucas^2^, Pedro Parreira^3^, Maria João Freitas^4^, Teresa Neves^3^, Elizabete Paz^5^, Anabela Salgueiro-Oliveira^3^

#### ^1^University of Coimbra, Coimbra, Portugal; ^2^Universidad de Extremadura, Extremadura, Spain; ^3^Health Sciences Research Unit: Nursing (UICISA:E) Nursing School of Coimbra, Coimbra, Portugal; ^4^Nursing School of São Francisco das Misericórdias, Lisbon, Portugal; ^5^Nursing Faculty Anna Nery, University of Rio de Janeiro, Minas Gerais, Brazil

**Correspondence:** Lisete Mónico lisete.monico@fpce.uc.pt

**Introduction**

In the present study we tried to achieve an understanding about the inducing agents of Stress and their effects on the employees of national organizations, analyzing the extent to which Emotional Intelligence and the Psychological Capital can function as protection factors of Organizational Stress

**Objective**

To verify into what measure Emotional Intelligence and Psychological Capital may constitute protection factors of Organizational Stress.

**Method**

The sample is composed of 301 employees, male and female of national organizations, aged between 18 and 67 and with diverse academic qualifications and organizational functions. As measures we used a questionnaire composed by organizational stress measures (self prepared), Emotional Intelligence (Rego et al., 2007) and Psychological capital (Luthans et al., 2007). We analyzed the construct of Emotional Intelligence as a repertoire of acquired psychological skills and we explored its contributions facing the challenges in the organizational field. We considered as POB states (Positive Organizational Behaviour) hope, resilience, trust and optimism, and the relations between Stress and Emotional intelligence.

**Results**

We found empirical support to the three hypothesis of Study 2: Hypothesis 4 – Organizational Stress correlates negatively with emotional intelligence and with the psychological capital of individuals; Hypothesis 5 – Emotional intelligence and psychological capital are positively correlated, and Hypothesis 6 – Emotional intelligence and psychological capital act as protection factors of Organizational Stress. We also analyzed the relations of Organizational Stress, of Emotional Intelligence and of Psychological Capital with a set of organizational and socio demographic variables.

**Discussion and conclusion**

The results were discussed considering the main generating sources of Organizational Stress and the way as Emotional Intelligence and Psychological Capital may constitute protection factors of the referred stress in employees.

**Keywords**

Organizational stress, emotional intelligence, psychological capital.

## A69 Psychological capital and emotional intelligence as protection factors in stress: what specificities in health professionals?

### Rita Rebola^1^, Lisete Mónico^2^, Paula Krepser^3^, Maria Felício^4^, Sheila Guagnini^5^, Anabela Salgueiro-Oliveira^1^, Pedro Parreira^1^

#### ^1^Health Sciences Research Unit: Nursing (UICISA:E) Nursing School of Coimbra, Coimbra, Portugal; ^2^University of Coimbra, Coimbra, Portugal ^; 3^Federal University of Juiz de Fora, Minas Gerais, Brazil; ^4^Higher School of Management Technology of Águeda (ESTGA), Aveiro University; ^5^Nursing Faculty Anna Nery, University of Rio de Janeiro, Minas Gerais, Brazil

**Correspondence:** Rita Rebola (ritah_rebola@hotmail.com)

**Introduction**

Empirical studies show that Psychological Capital has a predominant role in organizational practice leading to an increase in organization productivity and employee satisfaction. In addition Emotional Intelligence has been shown to have a positive impact on people management, with greater influence in health care.

**Objective**

This study intends to evaluate the relation and contribution of the psychological capital and Emotional Intelligence in the stress of the workers, especially those working in the health sector.

**Method**

An empirical, cross-sectional and non-experimental study was carried out. A non-probabilistic sample constituted of 874 workers, belonging to different professional groups (154 workers from the health care) and on whom a questionnaire was performed consisting of three scales: Scale of the Psychological capital (PC) (Luthans, Youssef & Avolio, 2007), Scale of Emotional Intelligence (EI) (Rego, Sousa, Pina e Cunha, Correia, & Saur-Amaral, 2007) and scale of Perceived Stress (PS) (Chioda, Dias, Silva, Marôco & Duarte, 2013). All the scales used showed good indexes of adjustment.

**Results**

The results suggest the existence of a highly positive relationship between the Emotional Intelligence and the Psychological Capital (R=.56), and a negative relation of moderate effect between the Emotional Intelligence and Stress (R=-.40) as well as a strong effect between the Psychological Capital and Stress (R=-.47) in workers of other professional categories. For the sample of health workers, the values increase slightly for the relation between the Emotional Intelligence and the Stress (R=-.49) and between the Psychological Capital and Stress (R=-.54). In addition, the hierarchical multiple regression analysis allowed us to identify that the Psychological Capital negatively predicts Stress in R2=22.4% in other professional categories and R2=29% in the health workers, and these values increase when the predictive influence of the Emotional Intelligence is added in ∆2=4.6% in health workers and only ∆2=2.5% in the other professional categories.

**Conclusion**

Psychological Capital and Emotional Intelligence present a negative relation in Perceived Stress with a greater magnitude in the health workers.

The results have a high importance in the management of organizations, namely in recruitment and professional training. Future research may be carried out to study the effect on organizations of the implementation of programs to increase Emotional Intelligence and Psychological Capital in teams.

**Keywords**

Psychological capital, emotional intelligence, perceived stress, people management

## A70 Emotional intelligence and workaholism

### Lisete Mónico^1^, Manuel Lopes^2^, César Fonseca^2^, Fábio do Prado^3^, Cristina Arreguy-Senna^4^, Paulo Pinto^4^, Pedro Parreira^5^

#### ^1^University of Coimbra, Faculty of Psychology and Education Sciences, Coimbra, Portugal; ^2^University of Évora – Nursing School of Évora, Évora, Portugal; ^3^University Center Dinâmico das Cataratas, Foz Iguaçu, Brazil; ^4^Federal University of Juiz de Fora, Minas Gerais-Brazil; ^5^Health Sciences Research Unit: Nursing (UICISA:E) Nursing School of Coimbra, Coimbra, Portugal

**Correspondence:** Lisete Mónico (lisete.monico@fpce.uc.pt)

**Introduction**

Emotional Intelligence concerns to a subject's competence in intelligent adaptive behaviour. It comprises the subjects’ capability to resort to complex information processing systems about their and others’ emotions, extended to the aptitude to use this data to monitor individuals’ reasoning and behaviour.

**Objective**

The question that arises in this research concerns to the relationship between emotional intelligence and Workaholism. Workaholism refers to spending large time working and the negative implications for workaholics’ social and family lives. It affects almost 1/4 of the worker population.

**Method**

A quantitative survey was made with a sample of individuals of both genders and different age groups that are currently employed in Portugal. Participants answered to a Workaholism Battery and an Emotional Intelligence Scale. Confirmatory factor analysis and Cronbach’s alpha were calculated, sustaining measurements’ validity and reliability. Cluster analysis was performed with the WorkBAT’s dimensions (Pleasure, Impulse, and Involvement) in order to found worker profiles.

**Results**

The cluster analysis suggest the existence of eight worker profiles - Work enthusiasts, Enthusiastic addicts, Work addicts, Disenchanted workers, Relaxed workers, Reluctant hard workers, Unengaged workers, Alienated professionals -, significantly affecting Emotional intelligence, *F*(7,287) = 4.75, *p* < .001, partial eta squared = .104. Workers inside the Work enthusiasts profile showed significant more Emotional Intelligence, followed by the Enthusiastic addicts and contrasting with the Unengaged workers, Disenchanted workers, and Relaxed workers, who received the lowest scores.

**Conclusions**

The most adaptive worker profiles are significantly related to higher levels of emotional intelligence. Understanding of own emotions, self-control against criticism, self-encouragement (use of emotions), emotional self-control (emotional regulation), empathy and emotional contagion, and understanding the others emotions are dimensions of Emotional intelligence that promote healthy workers’ profiles.

**Keywords**

Emotional intelligence, workaholism, worker profiles

